# Supplementary material for: Exploring five types of beam shaping using tiled-aperture coherent beam combining
Source: Commun Eng. 2025 Dec 2;5:7. doi: 10.1038/s44172-025-00562-8 (PMC12780195; doi:10.1038/s44172-025-00562-8)
Supplement: Supplementary file 2 — Supplementary Information [file 44172_2025_562_MOESM2_ESM.pdf]

Exploring five types of beam shaping using tiled-aperture coherent beam combining: Supplementary Information

A. Normalised power-in-bucket measurement and radii of simulated fibre outputs

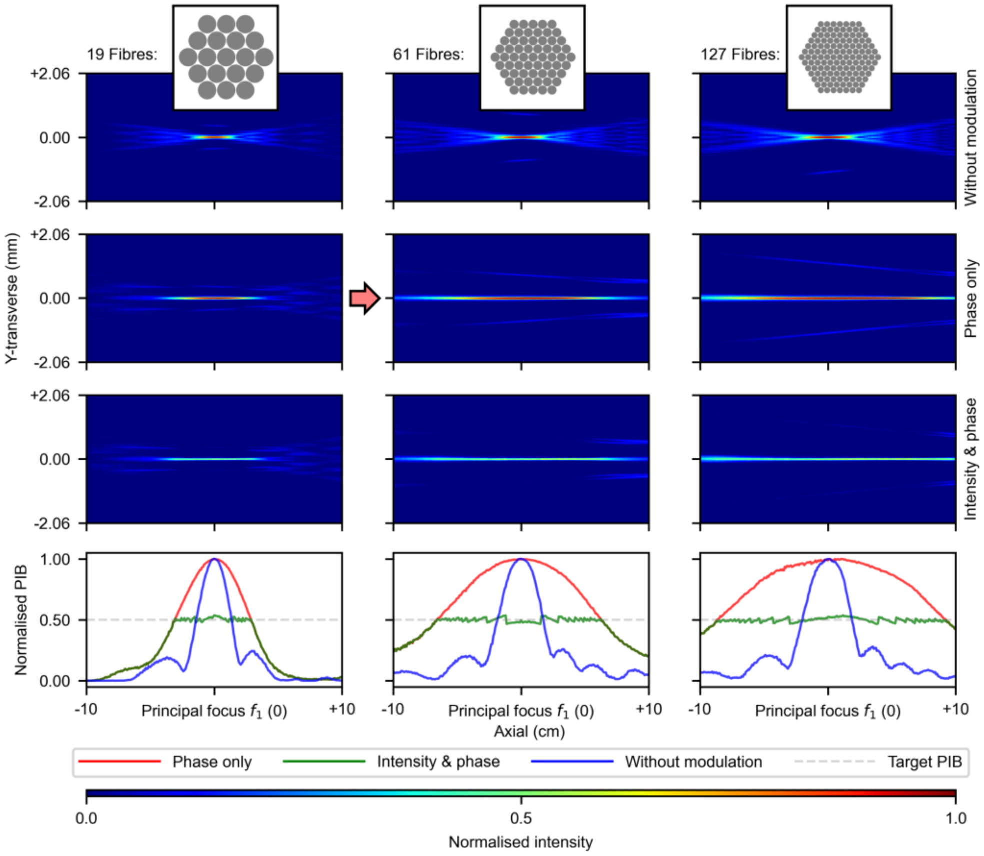

Fig. S1. Power-in-bucket measurements for 19, 61, and 127 simulated beamlets, with radii of the beamlets being 84, 42, and 28 SLM pixels, respectively.

In the Method Section, it has been shown that for a beam centred at (x^i, y^i) with phase φ^i, the phase profile after an ideal thin lens can be approximated by: $\phi_{out}(x,y) = Angle[T_{lens}(x,y) \cdot \exp(i \cdot \varphi^i)]$ subject to $(x - x^i)^2 + (y - y^i)^2 \leq r^2$. The phase at the centre of the beam geometry, which is given by: $\phi_{out}(x^i, y^i) = Angle[T_{lens}(x^i, y^i) \cdot \exp(i \cdot \varphi^i)]$, is used as the phase φ^i of this beam. Due to the quadratic term in the phase transformation function: $T_{lens}(x,y) = \exp\left[-i \cdot \frac{k}{2f} \cdot (x^2 + y^2)\right]$, the phase variation increases with the distance of the beam, centred at (x^i, y^i), from the centre of the lens. Beyond a certain distance, $\phi_{out}(x^i, y^i)$ no longer sufficiently approximates $\phi_{out}(x,y)$ within the area defined by $(x - x^i)^2 + (y - y^i)^2 \leq r^2$. However, due to the well-behaved nature (mainly continuous and monotonic) of the quadratic term $(x^2 + y^2)$, it is always possible to find a smaller beam radius $r_{small} < r$ such that the phase variation within the region $(x - x^i)^2 + (y - y^i)^2 \leq {r_{small}}^2$ remains sufficiently small, thereby allowing $\phi_{out}(x^i, y^i)$ to approximate $\phi_{out}(x,y)$ over this reduced area. Figure S1 visualises this effect, presenting Power-In-Bucket (PIB) measurements

and intensity distributions along the axial direction for 19 beams ($M^2 \approx 1.634$, 84 SLM pixel radii), 61 beams ($M^2 \approx 1.626$, 42 SLM pixel radii), and 127 beams ($M^2 \approx 1.482$, 28 SLM pixel radii), all with the same fill factor. The analysis compares these layouts without phase modulation and with phase-only focus steering along the axial direction. At each axial position, the three CBC layouts sample phases φ^i over approximately the same region of the lens phase profile, whilst layouts with a higher number of beamlets exhibit a smaller phase sampling interval due to their reduced radii. The results show that as the beamlet radii decrease, which allows $\phi_{out}(x^i, y^i)$ to more closely approximate the phase $\phi_{out}(x, y)$ within the region $(x - x^i)^2 + (y - y^i)^2 \leq r^2$, the phase-only modulation is capable of steering the combined beam focus farther from the principal focus of f_1. The beamlet radii, fill factor (i.e., sampling interval), and the number of beamlets (i.e., aperture size) all play a role in the beam focus steering. Their selection should align with specific application requirements, which are beyond the scope of this work and will not be discussed further.

In addition to the phase modulation for axial steering of the combined beam focus, intensity modulation can be also applied, as discussed in the Method section, to enable the PIB of the combined beam focus to remain constant during axial steering. Figure S1 demonstrates this effect, with the PIB truncated at 50% of the maximum. The intensity modulation described in the Method section adaptively reduces the intensity of each beamlet in the far field, homogenising the intensities across all beamlets. Beyond this per-beam modulation, a per-axial intensity adjustment is applied here at each axial position, uniformly reducing the intensities of all beamlets to maintain the PIB at 50% of the maximum if it exceeds this threshold. The observed blazed-like pattern arises from the binary grating, which can only modulate intensity quasi-continuously due to the limited SLM pixels available. The total time required to calculate the phases for 19, 61, and 127 beams, generate the corresponding patterns, and apply them to the SLM is measured to be 0.070 ± 0.004, 0.086 ± 0.004, and 0.1150 ± 0.007 seconds, respectively.

B. Simultaneous approximation of decentred lens and wedge prism in opposite tilting direction for beam focus steering along the transverse directions

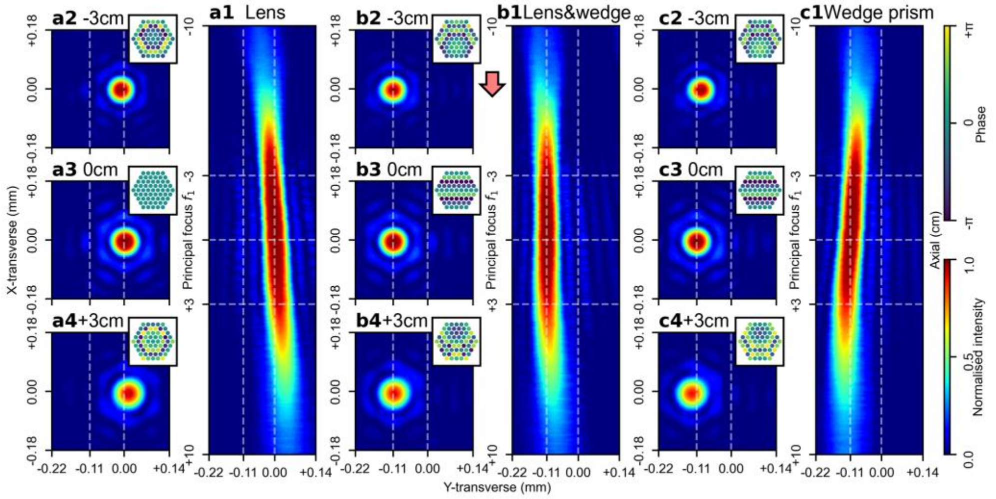

Fig. S2. **(a1)**, Intensity distribution along the axial direction by approximating the decentred second lens f_2, with transverse intensity distribution 3 cm before, at, and 3 cm after the principal focal plane of f_1 presented at **(a2)**, **(a3)**, and **(a4)**, respective. **(c1)**, Intensity distribution along the axial direction by approximating the second lens f_2 without any lens decentring, with transverse intensity distribution 3 cm before, at, and 3 cm after the principal focal plane of f_1 presented at **(c2)**, **(c3)**, and **(c4)**, respective. Instead, the phase profile of beams before the approximated lens f_2 is planar and linear, similar to the phase delays imposed by a wedge prism. **(b1)**, Intensity distribution along the axial direction by simultaneously approximating both the decentred second lens f_2 and the wedge prism, with transverse intensity distribution 3 cm before, at, and 3 cm after the principal focal plane of f_1 presented at **(b2)**, **(b3)**, and **(b4)**, respective.

As discussed in the Results section, both the wedge prism and decentred lens induce displacement of the combined beam focus along the transverse directions. The difference between these two approaches lies in the tilting direction: the wedge prism, which imposes planar, linear phase delays, diverges the combined beam focus immediately after leaving the first lens f_1. In contrast, the decentred lens converges the combined beam focus towards the principal focus of f_1 along the transverse directions after passing through the first lens f_1. By approximating the combination of a decentred lens and a wedge prism as a single optical element, the tilt state of the combined beam focus along the transverse directions can be steered with a higher degree of freedom. Figure S2 provides an example where the two approaches are applied simultaneously with opposite tilt directions, allowing the combined beam focus to remain stationary relative to the principal focus of f_1 along the transverse directions.

C. A different example of steering shaped beam focus in both axial and transverse directions

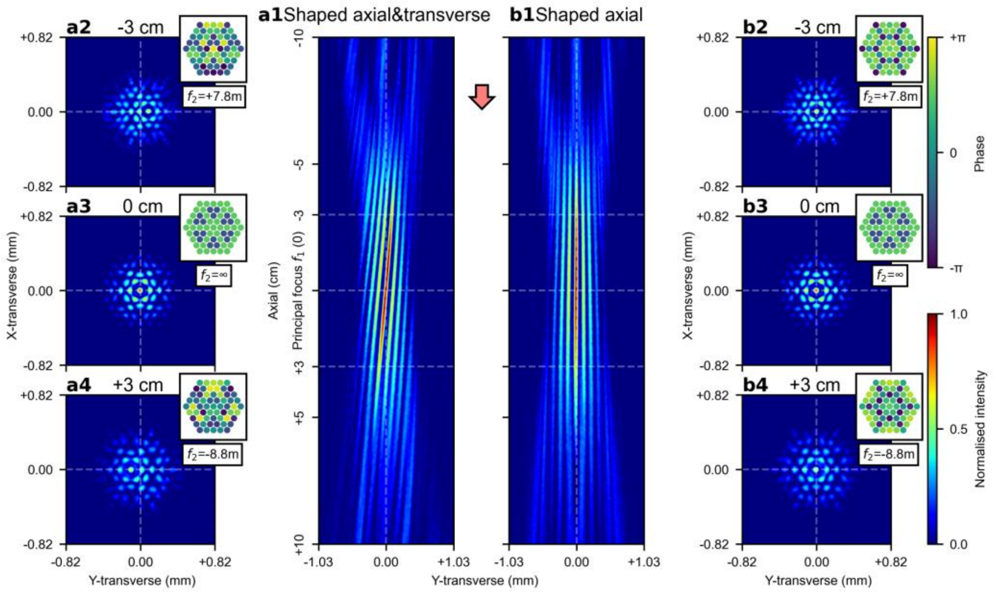

Fig. S3. **(a1)**, Simultaneous steering of a shaped beam focus in both the axial and the transverse directions. The transverse intensity distributions at three axial positions, 3 cm before, at, and 3 cm after the principal focal plane of f_1, are shown in **(a2)**, **(a3)**, and **(a4)**, respectively. **(b1)**, Axial-only steering of the same shaped beam focus. The transverse intensity distributions at the same axial positions are shown in **(b2)**, **(b3)**, and **(b4)**, respectively.

Figure S3 provides an additional example, supplementing Figure 5 in the Results section, demonstrating that a shaped beam focus can be simultaneously steered along both the axial and transverse directions.

D. Generating vortex beam via approximating diffractive spiral axicon

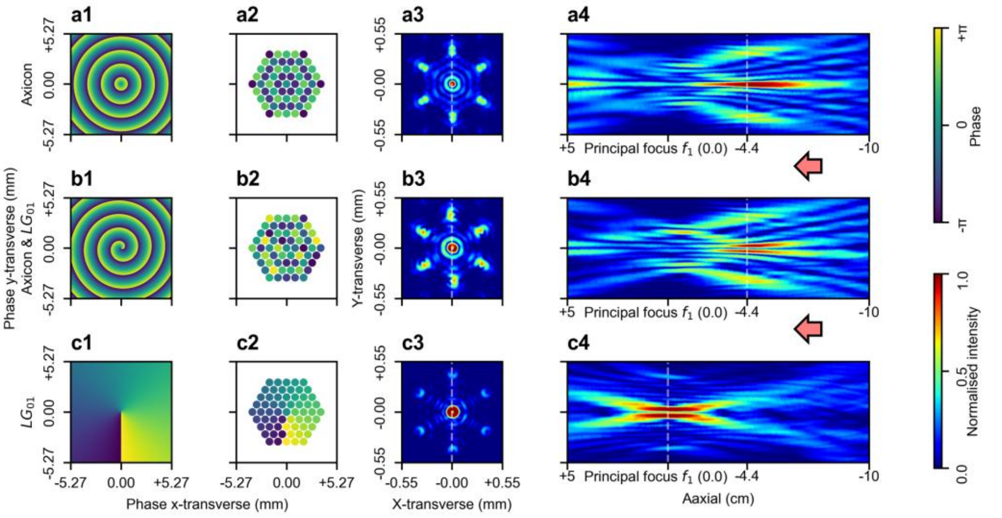

Fig. S4. **(a1)**, Phase delay imposed by an axicon lens, which can be approximated by offsetting the relative phase of beamlets, as shown in **(a2)**. **(a4)**, Intensity distribution along the axial direction resulting from **(a2)**, with the transverse intensity distribution 4.4 cm before the principal focus of f_1 shown in **(a3)**. **(c1)**, Phase delay along the azimuthal direction imposed by an LG_{01} spiral phase plate, approximated by the relative phase offsets of beamlets shown in **(c2)**. **(c4)**, Intensity distribution along the axial direction resulting from **(c2)**, with the transverse intensity distribution at the principal focus of f_1 shown in **(c3)**. **(b1)**, Phase delay imposed by a diffractive spiral axicon, similarly approximated by the relative phase offsets of beams shown in **(b2)**. **(b4)**, Intensity distribution along the axial direction resulting from **(b2)**, with the transverse intensity distribution 4.4 cm before the principal focus of f_1 shown in **(a3)**.

Following the discussion of orbital angular momentum in the Results section, Figure S4 demonstrates the generation of a vortex beam by simultaneously approximating both an axicon lens and a spiral phase plate, effectively approximating a diffractive spiral axicon.

E. Determining topological charges interferometrically with a planar-wave reference beam

The azimuthal phase delays of the combined beam foci, which carry OAM in different modes through the relative phase offsets between beamlets, are verified interferometrically using a planar-wave reference beam. This reference beam interferes off-axis with the combined beam focus, producing planar, linear phase differences between the two beams. In the absence of intensity singularities introduced by OAM modes, the interference pattern exhibits a comb-shaped interference pattern (i.e., evenly spaced linear fringes), as shown in Figure S5(a3). However, when intensity singularities associated with OAM modes are presented, these singularities disrupt the comb-shaped fringes, causing them to merge or split, resulting in characteristic fork-shaped interference patterns. Figure S5(b), Figure S5(c), and Figure S5(d) present the fork-shaped interference patterns for positive topological charges $l = 1$, $l = 2$, and $l = 3$, respectively, with a radial index p=0, where Figure. S5(e), Figure. S5(f), and Figure S5(g) present the fork-shaped interference patterns for negative topological charges $l = -1$, $l = -2$, and $l = -3$ outside and $l = 1$, $l = 2$, and $l = 3$ inside, respectively, with a radial index $p = 1$. The orientation of the fork-shaped fringes determines the sign of the topological charge: prongs pointing upwards indicate positive charges, whilst prongs pointing downwards indicate negative charges. Additionally, the number of forks corresponds to the absolute value of the topological charge (i.e., $|l|$). By combining these two characteristics, namely the orientation and number of forks, the complete information regarding the topological charge of the OAM-carrying combined beam focus is obtained.

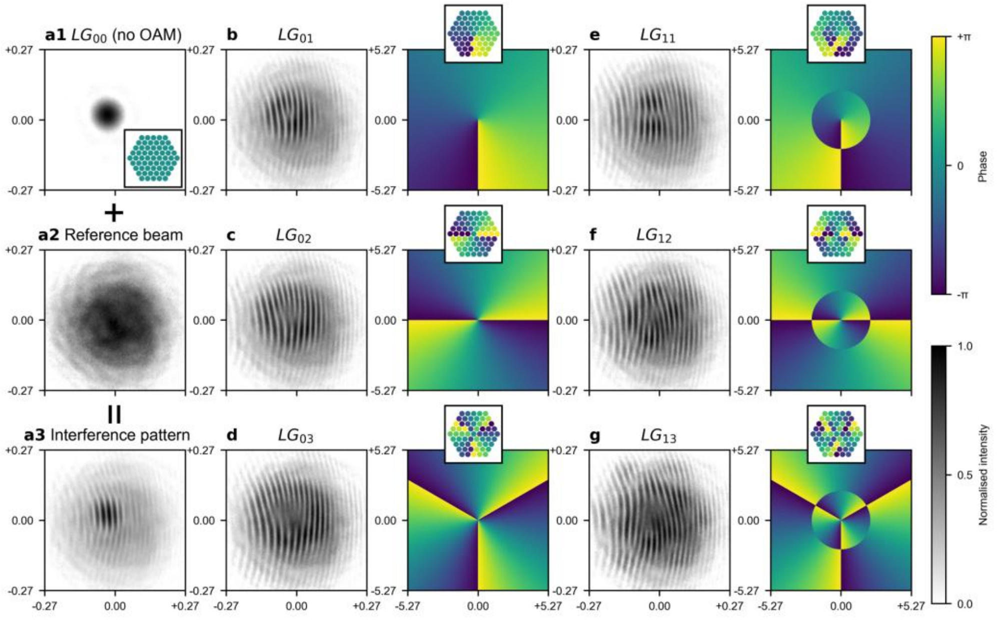

Fig. S5. **(a1)**, The combined beam focus, in the absence of relative phase differences between beams, corresponds to a beam in the LG_{00} mode, characterised by the lack of azimuthal phase variations. **(a2)**, A reference plane wave, incident at a slight angle relative to the modulated beam without any charge shown in **(a1)**, introduces planar, linear phase differences, resulting in the comb-shaped interference pattern shown in **(a3)**. On the other hand, when the reference beam interferes with beams that carry azimuthal phase delays, distinct fork-shaped interference patterns emerge; **(b)**, **(c)**, and **(d)** present fork-shaped patterns (left) with positive topological charges $l = 1$, $l = 2$, and $l = 3$, respectively, with a radial index $p = 0$ (right). Similarly, **(e)**, **(f)**, and **(g)** present fork-shaped patterns (left) with negative topological charges $l = -1$, $l = -2$, and $l = -3$ outside and $l = 1$, $l = 2$, and $l = -3$ inside, respectively, with a radial index $p = 1$ (right).

F. Axial and transverse tuneability for LG_{01}, LG_{02}, and LG_{03} OAM beams via approximating spiral phase plates and lenses simultaneously

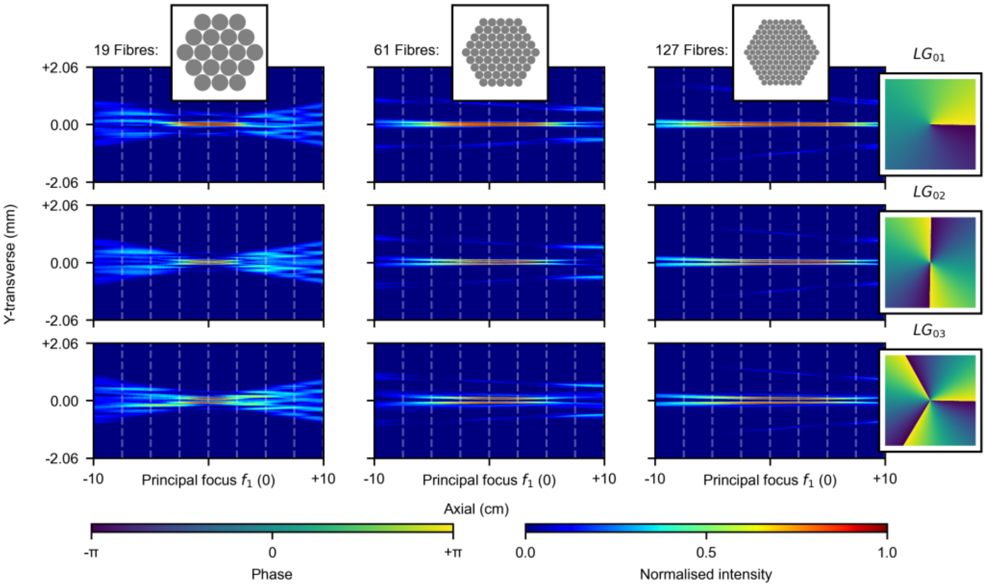

Fig. S6. Intensity distributions along the axial direction of three different OAM modes generated by arrays of 19, 61, and 127 beamlets with radii of 84, 42, and 28 pixels, respectively (so that all aperture sizes are approximately equal). Rows correspond to increasing array size (from 19 to 61 to 127 beamlets, top to bottom), and columns correspond to the LG_{01}, LG_{02}, and LG_{03} modes (left to right).

By approximating the combined phase profile of a spiral phase plate and a lens, the axial position of the focal point of an OAM-carrying beam can be controlled, as discussed in the Results section. Figure S6 presents additional examples of axial intensity distributions for the LG_{01}, LG_{02}, and LG_{03} modes, each approximated using arrays of 19, 61, and 127 beamlets with approximately equal aperture sizes. As shown in Figure S6, increasing the number of beamlets, whilst keeping the aperture size approximately the same, extends the axial steering range of the OAM beam. In addition, as (the absolute value of) the topological charge l increases, the axial steering range of the OAM beam becomes more limited. This observation is consistent with the discussion presented in Section A, which describes how increased phase variation (arising from a reduced focal length of the approximated lens) prevents the piston phase of a beamlet from accurately approximating the continuous phase profile within its own spatial extent. To maintain sufficient approximation under stronger phase variation, a smaller beamlet radius is required. Different from the discussion made in Section A, where the increased phase variation arises solely from modulating the focal length of approximated lens, the shown OAM beams involve additional phase variations in the azimuthal directions due to the higher topological charges l. Moreover, increasing the radial index p would further intensify phase variation, leading to similar limitations. However, for simplicity, the effects of increasing the radial index p are not included here. The intensity distributions along the transverse direction at axial positions of –10.0, –7.5, –5.0, –2.5, 0, 2.5, 5.0, 7.5, and 10.0 cm are shown in Figure S7.

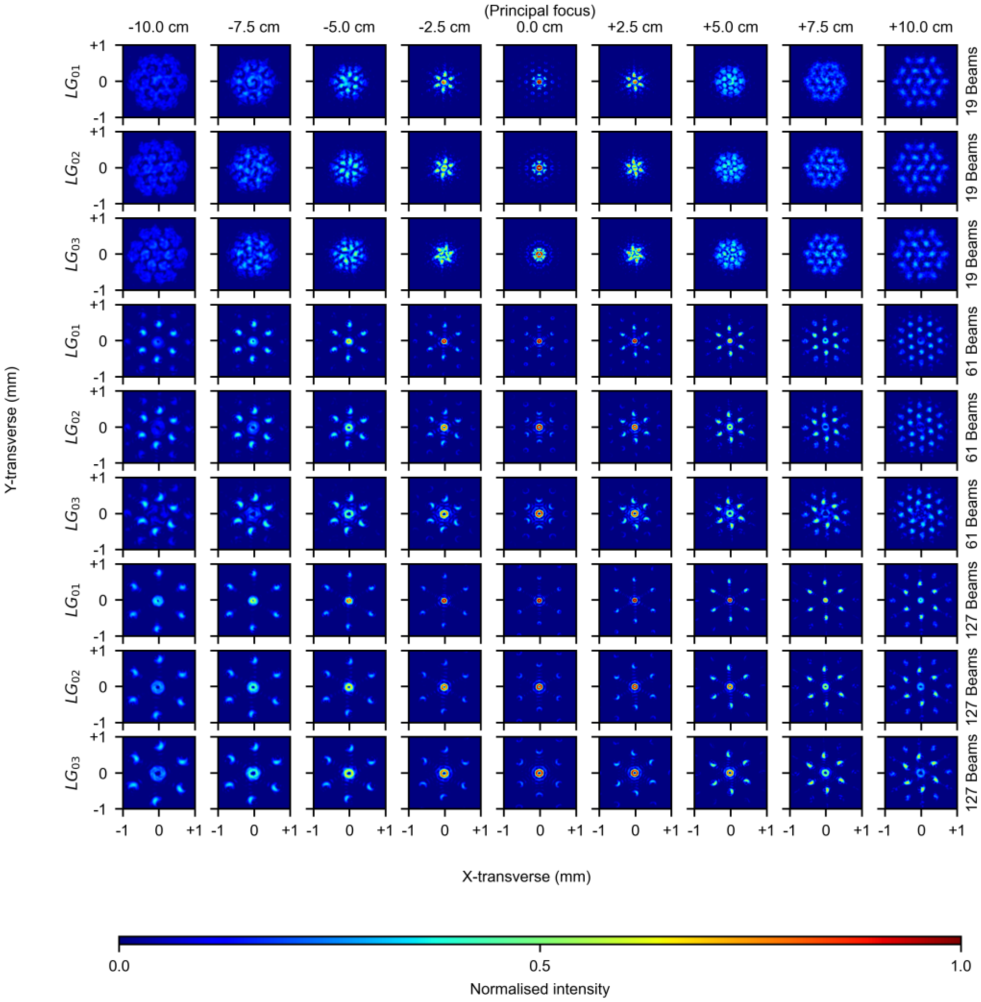

Fig. S7. Intensity distributions along the transverse direction of beams carrying the LG_{01}, LG_{02}, and LG_{03} modes, each approximated by arrays of 19, 61, and 127 beamlets. Intensity distributions are shown at axial positions of –10.0, –7.5, –5.0, –2.5, 0, 2.5, 5.0, 7.5, and 10.0 cm.

The transverse tuneability for the beams carrying LG_{01} mode is demonstrated in Figure S8, where, at progressively decreasing axial positions, an increasing lens decentring is applied. This allows transverse steering of the beams carrying LG_{01} mode along the x-axis at multiple axial positions. As detailed in the Results section, the effective focal length of the second lens, f_2, tends to infinity (i.e., the lens behaves as a plane-parallel plate) when the combined beam focus is axially steered to the principal focus of lens f_1. Consequently, decentring lens f_2 at this principal focus results in no transverse steering of the combined beam focus, as observed in the first row of Figure S8. Here, increased lens decentring progressively distorts the beam profile without shifting its position along the x-axis. Furthermore, Figure S8 also demonstrates that as the beam is steered axially away from the principal focus of f_1, identical amounts of lens decentring result in progressively larger transverse displacements of the combined beam focuses. This observation aligns with the results discussed in the Results section. Conceptually, lens decentring can be interpreted as introducing an angular tilt to the beam relative to the principal focus of f_1. The maximum achievable tilt angle (without visually distorting the intensity distribution) for the beam carrying LG_{01} mode and the corresponding beamlet array

characterised in Figure S8 is measured to be approximately 0.190°. A more advanced method of combined beam steering, involving the simultaneous approximation of the phase profiles of both a decentred lens and a wedge prism, has been demonstrated previously in Section B.

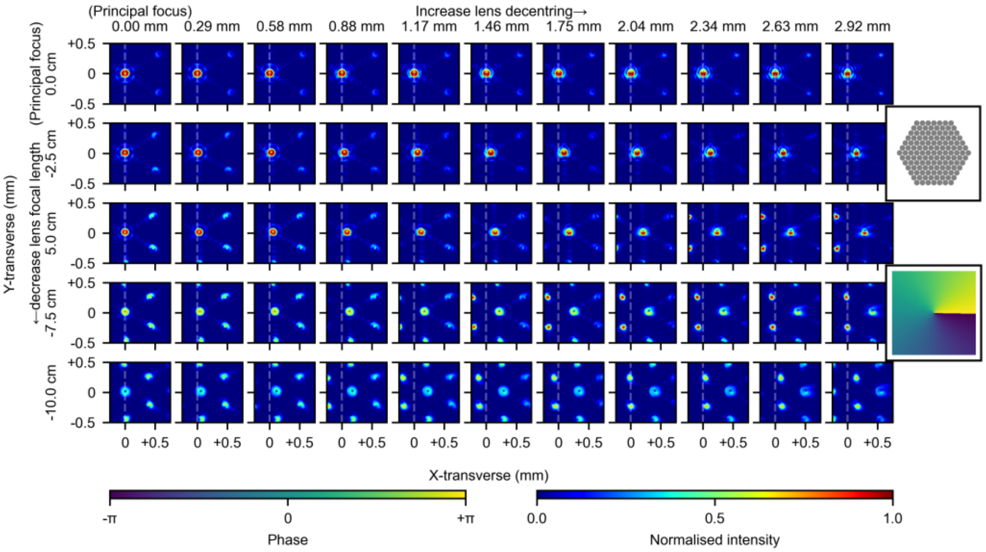

Fig. S8. Intensity distributions along the transverse direction of beams carrying the LG_{01} mode, approximated by an array of 127 beamlets (radii of 28 pixels). Each column shows the intensity distributions at a decreasing axial distance (0.0, -2.5, -5.0, -7.5 and -10.0 cm from top to bottom), whilst each row shows the intensity distributions at the same position with an increasing lens decentring (from 0.00 mm to 2.92 mm, left to right).

Figure S9 demonstrates the transverse tuneability for the beams carrying LG_{01}, LG_{02}, and LG_{03} modes at an axial position -7.5 cm from the principal focus of lens f_1, each mode approximated by the same beamlet array (127 beamlets with 28-pixel radii). It is evident that as the topological charge l increases, transverse tuneability decreases. Specifically, at higher lens decentring values, the resultant intensity distribution progressively deviates further from the ideal ring-like intensity distribution (from OAM mode). This decrease in transverse tuneability with increasing topological charge l is similar to the previously observed decrease in axial tuneability; this occurs because higher topological charges l introduces greater phase variations, making piston phase less effective at approximating the continuous phase profile within each beamlet's spatial extent. The maximum achievable tilt angles (without significant visual distortion of the ring-like intensity distribution) measured for the beams carrying LG_{01}, LG_{02}, and LG_{03} modes in Figure S9 are approximately 0.190°, 0.159°, and 0.096°, respectively.

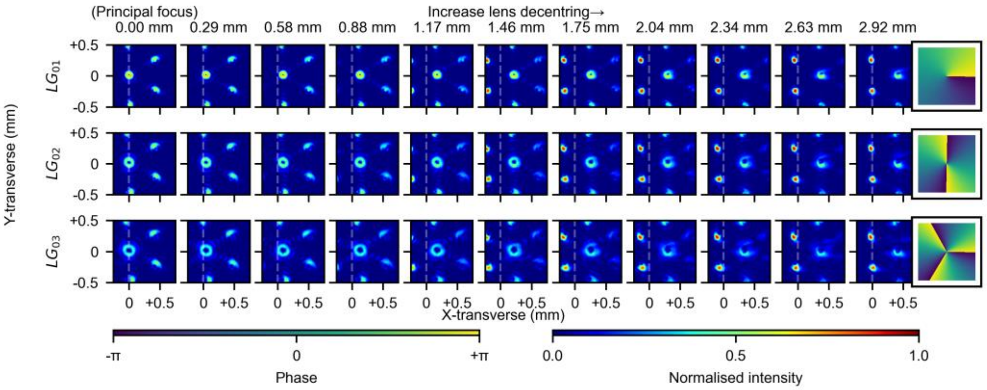

Fig. S9. Intensity distributions along the transverse direction of beams carrying the LG_{01}, LG_{02}, and LG_{03} mode (first, second, and third row, respectively), each approximated by an array 127 beamlets (radii of 28 pixels). All intensity distributions are presented at the axial position of -7.5 cm from the principal focus of the lens f_1, and each row illustrates the effect of increasing lens decentring, progressing from 0.00 mm to 2.92 mm (left to right).

Another critical factor affecting the transverse tuneability of beams carrying OAM modes is the aperture size of the beamlet array. Significant lens decentring can displace the phase singularity (i.e., the vortex core) beyond the restricted aperture of the beamlet array, leaving only residual fringe-like phase patterns at axial positions away from the principal focus of lensf_1. This condition results in a spot-like intensity distribution with transverse displacement, effectively approximating a wedge prism. Figure S10 demonstrates the transverse tuneability of beams carrying the LG_{01} mode, approximated by beamlet arrays of progressively larger aperture sizes achieved by increasing the number of beamlets (from 19 in the first row to 61 in the third row, and 127 in the fifth row), whilst maintaining a constant beamlet radius. It can be observed that with smaller aperture sizes, the characteristic ring-like shape of the LG_{01} mode deteriorates into a single spot as the phase singularity exits the limited aperture. Conversely, expanding the aperture size by increasing the number of beamlets improves transverse tuneability by maintaining the phase singularity within the aperture, even at higher lens decentring values.

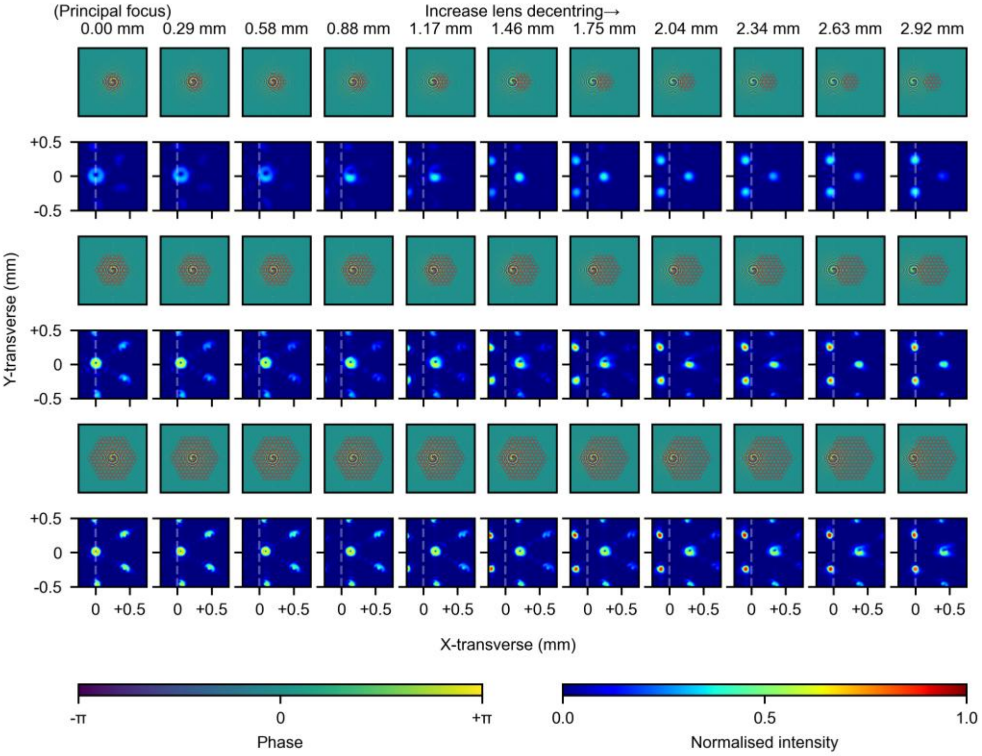

Fig. S10. Intensity distributions along the transverse direction of beam carrying the LG_{01} mode, approximated by arrays of 19, 61, and 127 beamlets (first, third, and fifth rows, respectively); each beamlet has an identical radius of 28 pixels, leading to progressively increasing aperture sizes. The corresponding target phase profiles, which the beamlet arrays aim to approximate for simultaneous axial and transverse steering, are shown in the second, fourth, and sixth rows for the 19, 61, and 127 beamlet arrays, respectively, with outlines of the beam arrays overlaid on the phase profiles. All intensity distributions are presented at the axial position of -7.5 cm from the principal focus of the lens f_1, and each row illustrates the effect of increasing lens decentring, progressing from 0.00 mm to 2.92 mm (left to right).

From the discussions and results presented above, it can be concluded that the beam steering capability of the proposed phase control strategy is fundamentally governed by the physical characteristics of the beamlet array. Specifically, arrays with a higher number of smaller beamlets are preferable, as they facilitate combined beam focus steering across a larger space and support higher-order modes. However, devising a compact, high-power, and high-channel-count CBC system presents substantial engineering challenges. Overcoming these obstacles, including thermal management and phase noise suppression, will be the focus of our future research efforts.
