# Supplementary material for: Exploring five types of beam shaping using tiled-aperture coherent beam combining
Source: Commun Eng. 2025 Dec 2;5:7. doi: 10.1038/s44172-025-00562-8 (PMC12780195; doi:10.1038/s44172-025-00562-8)
Supplement: Supplementary file 3 — Description of Additional Supplementary Files [file 44172_2025_562_MOESM3_ESM.pdf]

Description of Additional Supplementary Files

File name- Supplementary Movie

File description – Phase-only five-dimensional control of a coherently combined beam: sequential x–y–z focus steering, azimuthal rotation of higher-order LG modes, and LG-mode switching.
